# Jurisdictional Comparison in the Utilization and Valorization of Animal By-Products of Slaughterhouse-Origin: A Global Review

**DOI:** 10.3390/foods15081324

**Published:** 2026-04-10

**Authors:** Ifedayo E. Bello, Tawanda Tayengwa, Julianne Roe, Jianping Wu, Olugbenga P. Soladoye

**Affiliations:** 1 Agriculture and Agri-Food Canada, Lacombe Research and Development Center, 6000 C and E Trail, Lacombe, AB T4L 1W1, Canada; ifedayo@ualberta.ca (I.E.B.); tawanda.tayengwa@agr.gc.ca (T.T.); 2 Department of Agricultural, Foods & Nutritional Science, Faculty of Agricultural, Life & Environmental Sciences, North Campus, University of Alberta, 116 St & 85 Ave, Edmonton, AB T6G 2R3, Canada; julianner921@gmail.com (J.R.); jwu3@ualberta.ca (J.W.)

**Keywords:** animal by-products, jurisdictional comparison, valorization, consumer preferences, circular bioeconomy, sustainability

## Abstract

Animal by-products (ABPs), comprising both edible and inedible components, offer significant nutritional, economic, and environmental value. However, their utilization differs markedly across global jurisdictions due to cultural preferences, regulatory frameworks, and technological capacities, which collectively shape consumption patterns and determine integration into food systems or diversion to industrial applications. While consumer reliance on offal remains high in the Global South, driven by tradition, affordability, and nutritional needs, its acceptance in the Global North is markedly lower, often limited by cultural aversion and perceived risks. Drawing from published evidence and primary survey data, this review examines regional consumption trends, industrial utilization pathways, and emerging valorization opportunities for ABPs. Globally, industrial use of ABPs is increasingly shifting toward advanced bioprocessing, integration within circular bioeconomy models, and high-value applications in nutraceutical, pharmaceutical, and bio-industrial sectors. An online cross-sectional survey (*n* = 358) conducted across Africa, North America, Europe, and Asia revealed strong regional disparities in offal consumption, with higher acceptance in parts of Africa and Asia and more selective use in Europe and North America. Respondents also indicated clear support for non-food valorization pathways, particularly animal feed, fertilizer, and energy production, alongside pharmaceutical and cosmetic applications. These findings align with the literature, where industrial valorization pathways such as collagen and gelatin extraction, rendering, and bioenergy production dominate. This review synthesized the jurisdictional disparities in consumption, regulation, technological capability, and industrial applications while highlighting emerging technological opportunities for high-value valorization. Recommendations emphasize consumer education, regulatory refinement, technological innovation, and sustainable practices to enhance the economic and environmental benefits of ABP utilization within a circular bioeconomy framework.

## 1. Introduction

In the context of modern global food systems, meat production plays a pivotal role in meeting the nutritional needs of a growing population. One inevitable outcome of rising meat output is the generation of substantial volumes of animal by-products (ABPs), which represent an underutilized resource with significant potential for value addition and sustainable use [1]. As global meat production continues to increase, projected to reach approximately 365 million tons in 2024 [2], the effective management of these by-products has become increasingly critical for minimizing environmental and social impacts and maximizing economic benefits.

ABPs include all parts of a slaughtered animal, whether edible or inedible, that do not form part of the dressed carcass [3,4]. The proportion relative to live animal weight varies by species, ranging from 30 to 44% in cattle and 20 to 30% in pigs [5,6]. Historically, the inclusion of these ABPs in human diets dates back roughly 2.6 million years, prior to the evolution of fire-based food preparation. During this period, soft tissue such as bone marrow, liver, kidneys and brain were preferred because they could be consumed raw due to their tenderness and chewability [7,8]. The advent of fire not only improved the palatability of nutrient-dense animal tissues but also enhanced their safety and digestibility, enabling broader consumption of mammalian avian, and aquatic flesh [9].

Global consumption of animal protein has increased steadily over recent decades and continues to rise. According to recent estimates, global meat consumption exceeded 360 million tons in 2022 and is projected to reach approximately 374 million tons by 2030, driven by population growth, urbanization, and rising incomes, particularly in developing regions. While growth rates have slowed in high-income countries, demand for beef, poultry, and pork remains strong globally, with poultry showing the fastest expansion in recent years [10]. These trends are driven by increasing demand for nutritious, protein-rich foods, facilitated by industrialization, urbanization and rising per capita income in many developing regions. Correspondingly, the volume of generated ABPs has grown. Traditionally, many of these ABPs were discarded through incineration, landfilling or other disposal routes [11] or at best rendered. However, such approaches are resource-intensive, environmentally burdensome, contributing to high biological oxygen demand in effluents and greenhouse gas emissions (GHGEs) from decomposition, and fail to capture the substantial inherent value of these materials [12]. These linear and inefficient models are increasingly viewed as unsustainable.

Despite their high potential, many by-products remain underexploited, even though they could contribute substantially to food, pharmaceutical, agricultural, and bio-industrial sectors [13,14]. This has contributed to a global shift toward circular bioeconomy models that emphasize the conversion of ABPs into higher-value co-products [15]. In line with this shift, growing emphasis on sustainability has intensified interest in using ABPs as renewable resources [1,16]. Such valorization strategies not only reduce waste generation and GHGEs but also enhance economic returns and resource efficiency across the meat value chain [17]. A rapidly expanding body of research highlights technological advances including rendering innovations, enzymatic hydrolysis, collagen extraction, and biorefinery integration that enable the conversion of low-value tissues into high-value products such as bioactive peptides, biopolymers, and biofuels [18,19]. This emerging paradigm positions ABPs as essential resources for advancing sustainable food systems, waste reduction, and industrial innovation.

However, despite these opportunities a major challenge remains the lack of harmonized frameworks and comparative analyses that consider jurisdictional differences in utilization practices, technological capacity, consumer acceptance, and regulatory contexts. These jurisdictional peculiarities strongly influence the positioning, value recovery, and marketability of by-products. Although numerous studies have examined traditional and emerging valorization technologies in specific regions, there is a notable gap regarding comprehensive comparative analyses that synthesize differences across jurisdictions. This knowledge gap hinders global efforts to design customized valorization strategies and maximizes resource recovery. Accordingly, a comprehensive review is needed to integrate existing knowledge on global utilization patterns, identify barriers and opportunities, and propose feasible pathways for enhanced valorization within circular bioeconomy models. Therefore, the objective of this review was to examine jurisdictional differences in consumer consumption patterns and industrial utilization of ABPs, across the Global North and South. It further aimed to explore emerging valorization opportunities within these jurisdictional realities and provide recommendations for sustainable practices to maximize the potential of these resources.

To achieve these objectives, this study adopts a hybrid approach that integrates a structured literature review with primary survey data. While existing literature provides extensive insights into technological advancements, regulatory frameworks, and historical utilization patterns of animal by-products, it often lacks real-time evidence on consumer perceptions and behavioral drivers across different jurisdictions. The inclusion of a cross-sectional survey therefore enables the generation of current empirical data on consumption patterns, preferences, and barriers, particularly across the Global North and Global South. This combined approach allows for direct comparison between documented trends and actual consumer responses, thereby strengthening the analytical depth of the study. Ultimately, integrating both secondary and primary data enhances the robustness of the findings and supports the development of more practical, evidence-based recommendations for sustainable valorization of animal by-products.

### Classifying Animal By-Products

In meat processing systems, maximizing the value of each animal extends far beyond the sale of the dressed carcass. ABPs can constitute up to 40% of an animal’s live weight, making their effective utilization essential for improving profitability. Historically, the dressed carcass alone has been insufficient to offset production costs, since the live animal often costs more than the resulting carcass [20]. This economic reality highlights why by-products previously accounting for as much as 15% of slaughterhouse income [21] have become increasingly important within modern processing operations. As global markets evolved, regulatory frameworks such as the European Commission’s (EC) 2009 regulations emerged to ensure safe handling and categorization of these materials [3].

To improve clarity, slaughterhouse-derived meat by-products can be broadly classified into edible and inedible categories based on their intended use and regulatory framework (Table 1). ABPs can be classified using several criteria, including edibility, appearance, jurisdiction considerations, risk level, and intended end use. Among these, edibility remains the most widely applied standard distinguishing by-products as either edible or inedible. However, these classifications differ across cultural, religious, socioeconomic, and regulatory contexts [22,23]. For instance, lungs are regarded as a delicacy in traditional Scottish cuisine but are considered inedible in the United States due to food safety regulations [24]. Edible by-products typically include organs such as liver, heart, kidney, and tongue, which may be consumed directly or after minimal processing [14,25]. In contrast, inedible by-products including rumen digesta, skin, horns, hooves, and bones are excluded from human consumption due to safety restrictions or cultural norms [10].

Another common method of classification is based on appearance, which groups offal into red, white, and green categories. Red offal consists of highly vascularized organs such as liver, kidney, spleen, tongue, and heart. White offal includes fatty or glandular tissues like brain, bone marrow, fat, and testicles. Green offal is derived from the digestive system, including the rumen and intestines [22,23,26,27]. In jurisdictions with risk-based regulatory frameworks, most notably the European Union, classification aligns with EC Regulation No. 1069/2009, which categorizes ABPs into three levels of risk. Category 1 represents the highest risk materials, including tissues potentially associated with transmissible spongiform encephalopathies (TSEs). Category 2 includes medium risk materials such as manure or condemned carcasses, while Category 3 comprises low risk materials deemed fit for human consumption but excluded from the food chain for other reasons [3,28].

Beyond biological and regulatory factors, social, cultural, and economic dynamics strongly influence how ABPs are classified and utilized. Religious practices, cultural traditions, and household income play significant roles in determining which by-products are accepted for consumption or used for alternatives applications [13]. Finally, classification can also be based on end-use functionality. ABPs serve diverse roles across industries, providing raw materials for agricultural inputs (such as bone meal and organic fertilizers), industrial goods (such as gelatin, glue, and casings), and pharmaceutical products (including insulin, pepsin, hormones, and bioactive compounds) [29]. These multidimensional classifications underscore the versatility and economic significance of ABPs across food and non-food sectors.

## 2. Proportion and Chemical Composition of ABPs Based on Edibility

**Figure 1:**
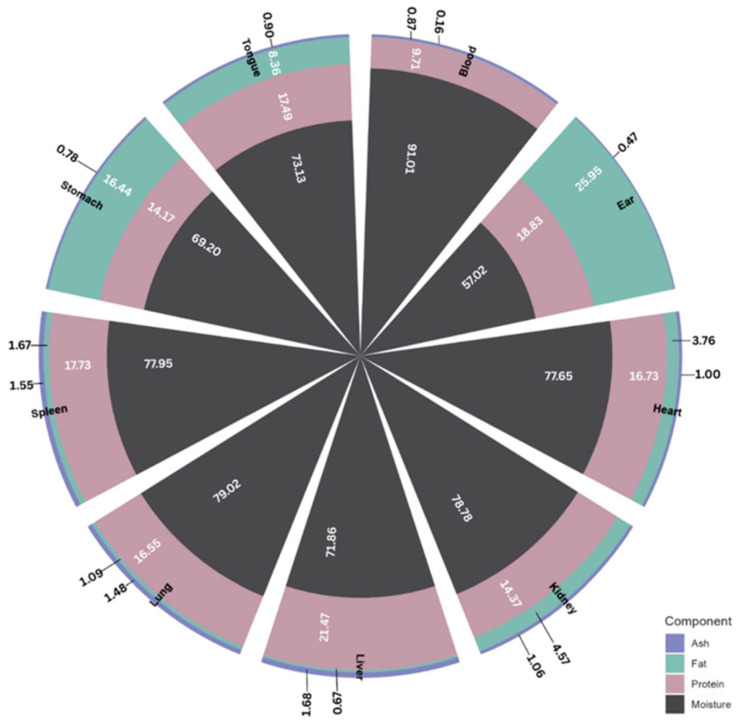
Chemical composition of selected pork offal. Adapted from [42].

Classifying ABPs according to edibility provides a clearer understanding of their nutritional and industrial value and aligns with global regulatory standards and cultural interpretations of what constitutes food [14,25]. The proportion and chemical composition of these materials vary widely and are influenced by several intrinsic and extrinsic factors including species, breed, age, diet, sex, and live weight [30,31]. Figure 1 shows the chemical composition of selected pork offal.

Edible offal typically accounts for approximately 10–15% of an animal’s live weight, with the exact proportion depending on species and breed [13,20]. These tissues are nutrient-dense, providing high-quality proteins, essential amino acids, a broad spectrum of vitamins (A, B-complex, and D), and critical trace elements such as iron, zinc, and selenium [12,32]. Specific organs offer distinct nutritional and functional benefits: liver and kidney are exceptionally rich in micronutrients and bioactive compounds, while lungs and tripe contain notable amounts of collagen and elastin [33,34]. Blood, constituting up to 4% of live weight, is especially valuable due to its source of plasma proteins, which possess desirable functional properties such as emulsification, foaming and water-binding capacity [35,36].

Despite their strong nutritional profile, the utilization of edible by-products varies significantly across regions. In many parts of Asia, Africa, and the Middle East, offal remains integral to traditional culinary practices. Conversely, in Western nations, consumption is comparatively low, constrained by cultural preferences, limited familiarity, and in some cases, regulatory restrictions [37]. Consequently, a large proportion of edible by-products in these regions is diverted to secondary uses such as animal feed, pet food formulations, or pharmaceutical processing [38,39]. Nonetheless, edible offal continues to play an important global dietary role and represents a promising avenue for improving food security and diversifying accessible protein sources. Inedible by-products, by contrast, encompass materials not typically consumed by humans but that serve as essential inputs for agricultural, industrial, and pharmaceutical applications. These include hides, skins, horns, bones, rumen digesta, hooves, and feathers, which collectively contribute approximately 5–15% of live weight depending on species and processing methods [13,40]. Hides and skins are particularly valuable due to their high collagen content and are primarily processed into leather or converted into gelatin and collagen hydrolysates for use in foods, cosmetics, and pharmaceutical products [33,41]. Bones, hooves and horns are commonly utilized in the production of fertilizers, animal feed mineral supplements, and various industrial products [29]. Rumen digesta, though often discarded, is gaining attention as a substrate for biogas production and composting, supporting waste reduction and contributing to environmental sustainability [38].

Overall, while edible by-products represent nutrient-rich yet underutilized food resources, and inedible fractions serve as economically significant raw materials for multiple industries, both categories offer substantial opportunities for value addition through targeted valorization strategies. Their diverse chemical composition and functional versatility underscore their importance within circular bioeconomy frameworks and set the foundation for examining emerging industrial and commercial valorization pathways in the next section.

## 3. Valorization Potentials for Animal By-Products in Industrial and Commercial Applications

The industrial valorization of ABPs has progressed far beyond traditional rendering, expanding into a diverse, multi-sectoral value chain that supports the food, pharmaceutical, cosmetic, agricultural, and bioenergy industries. This shift reflects a broader movement towards sustainable production systems guided by circular bioeconomy principles. As previously noted, global meat processing systems generate over 150 million tons of by-products annually [1], yet only a portion is currently transformed into high-value commodities. Increasing global production underscores the need for scalable industrial utilization strategies that enhance profitability, minimize waste, and contribute to sustainable development goals. The subsections below highlight major valorization pathways and their emerging potential.

### 3.1. Rendering and Fat Recovery

Rendering remains the foundational technology for converting ABPs into stable, value-added ingredients. Through thermal processing, fats, proteins, and minerals are separated from ABPs such as bones, trimmings, and visceral organs to produce products including meat and bone meal (MBM), tallow, lard, and related derivatives. In the United States and Europe, more than 50% of slaughterhouse by-products are rendered, contributing up to 15% of total slaughterhouse revenue [13]. Economically, these products represent approximately 11.4% of gross income from beef and 7.5% from pork [38], where over 25 million tons of ABPs are processed annually to supply feed, biodiesel, and oleochemical industries [42]. While conventional rendering relies on wet or dry thermal processing, emerging low-temperature enzymatic rendering technologies offer improved protein quality and significantly reduced GHGEs, strengthening the environmental sustainability of the sector [43]. As of 2022, Table 2 presents the current global rendering output by region.

**Table 2:** Estimated global rendering output by region (2022).

Region	Annual By-Product Volume Processed (Million Tons)	Main Outputs	Key Applications
**North America**	25.0	Tallow, MBM, poultry meal	Biodiesel, pet food, feed, oleochemicals
**Europe**	19.5	MBM, animal fats, blood meal	Fertilizer, feed, cement kiln fuel
**Asia-Pacific**	32.0	Poultry meal, fishmeal, lard	Aquaculture, livestock feed, industrial fats
**Latin America**	12.5	Bovine tallow, MBM	Soap, biodiesel, animal feed
**Africa**	5.5	Mixed rendered fats, MBM	Feed supplements, artisanal soap production

Source: Adapted from [44], MBM = Meat-and-Bone-Meal.

### 3.2. Use of Intestines and Stomachs as Natural Casings

A significant application of slaughterhouse-derived by-products is the use of intestines and stomach tissues as natural casings in sausage production. These materials, obtained primarily from cattle, sheep, and pigs, are processed through cleaning, grading, and preservation before use. Natural casings are highly valued due to their permeability, elasticity, and ability to enhance flavor and texture. Globally, the natural casing industry represents an important economic segment within the meat value chain, supporting both artisanal and industrial sausage production. Compared to synthetic alternatives, natural casings are often preferred in premium products due to their superior sensory characteristics. This application highlights the importance of gastrointestinal by-products as high-value resources within circular bioeconomy systems.

### 3.3. Gelatin and Collagen Extraction and Industrial Applications

Hides and skins generated during slaughter represent a major source of collagen, which can be processed into gelatin and bioactive peptides. These materials constitute one of the most valuable structural by-products due to their high protein content and functional versatility. In industrial processing systems, hides and skins are routinely collected, preserved, and subjected to pretreatment processes to facilitate collagen extraction. In the food sector, collagen-derived materials are widely used in edible films, stabilizers, and artificial casings for processed meat products such as sausages. Collagen casings, produced from reconstituted collagen extracted from hides, offer advantages in uniformity, mechanical strength, and scalability compared to natural casings. Their use has expanded significantly in industrial meat processing due to their consistency and suitability for automated production systems. In addition, collagen and gelatin are increasingly incorporated into functional foods due to their nutritional and health-related properties. Beyond food applications, collagen extracted from hides is extensively utilized in biomedical materials, wound healing products, pharmaceutical formulations, and cosmetic applications. These include tissue scaffolds, drug delivery systems, and skin-repair formulations. The recovery and utilization of collagen from hides and skins exemplify efficient resource use, contributing significantly to waste reduction and sustainable meat processing systems.

**Figure 2:**
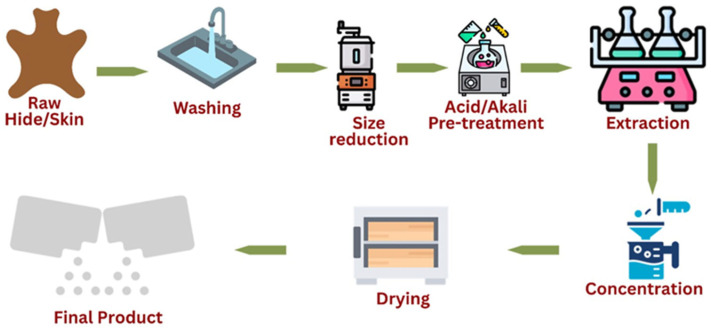
Process flow for collagen and gelatin extraction from meat by-products. Adapted from [50].

Gelatin derived from collagen in animal connective tissues has long been valued for its functional properties, including gelling, stabilizing, and emulsifying [14,45]. Hides, skins, and bones constitute the primary feedstocks for gelatin and collagen production, with global output estimated at approximately 620,000 tons annually [45]. The gelatin market continues to expand due to its diverse applications across food, pharmaceutical, nutraceutical, and cosmetic industries. Traditionally used in confectionery, dairy formulations, and processed meats, gelatin’s biocompatibility has facilitated its incorporation into pharmaceutical capsules, wound dressings, anti-aging products and controlled drug delivery systems [46]. Its hydrolyzed derivatives, marketed as collagen peptides, are widely used in nutraceuticals for joint, bone, and skin health. The cosmetics sector shows growing demand for gelatin-based ingredients, particularly in anti-aging formulations and skin-repair [47,48]. Production is achieved through acidic, alkaline [49], or enzymatic processes [50], each imparting distinct physicochemical properties that influence final product functionality [19]. Overall, gelatin extraction involves three major segments: pretreatment, extraction, and post-processing as shown in Figure 2.

### 3.4. Pharmaceutical and Biomedical Applications

Pharmaceutical and biomedical industries represent some of the highest-value valorization pathways. Blood-derived heparin primarily sourced from porcine lungs and intestines exceeds 100 tons in global annual production [51]. Other widely used biomedical derivatives include insulin from porcine pancreas and pepsin from stomach mucosa. Collagen from hides is a critical biomaterial for scaffolds used in tissue engineering and wound healing, with the biomedical collagen market projected to grow at a Compound Annual Growth Rate (CAGR) of 6–8% through 2030 [52]. In South Asia, hides also underpin a robust leather industry, with countries like India generating significant export revenues [53,54]. Enzymatic hydrolysis of organs such as livers and glands has further enabled the production of bioactive peptides exhibiting antioxidant, antihypertensive, and antimicrobial properties, supporting a rapidly expanding nutraceutical sector [18].

### 3.5. Blood Plasma Processing

Processing slaughterhouse blood enables recovery of valuable plasma proteins prized for their gelling, emulsifying, and water-binding properties [51,55]. These proteins significantly improve the texture, stability, and moisture retention of processed meat [17]. Beyond plasma, globin fractions serve as functional ingredients in nutritional and fortified foods because of their high protein content and bioactivity [56]. Plasma powders are widely used in processed meat products including sausages and hams to enhance juiciness and structural integrity [57]. France’s rendering network for example, recovers over 60,000 tons of blood annually for use in both human and pet food applications [55]. This pathway simultaneously reduces waste and strengthens protein circularity within the meat sector.

### 3.6. Pet Food Manufacturing

The pet food industry remains one of the largest consumers of ABPs, particularly edible offal, mechanically separated meat, and rendered protein meals. In the United States, more than 30% of rendered by-products are incorporated into dry and canned pet food [58,59]. These ABPs supply essential proteins, fats, vitamins, and minerals, while contributing to sustainability by diverting high-quality nutrients from landfill or low-value disposal [27,58]. Growing demand for premium pet foods has increased emphasis on traceability, ingredient transparency, and functional health claims, prompting more stringent quality assurance and certification standards across the value chain [60]. As a result, pet food manufacturing has become an important driver of innovation and high-value utilization of ABPs.

### 3.7. Agricultural Applications

ABPs also play a critical role in agriculture, particularly in livestock and aquaculture nutrition and in soil fertility management. Meat and bone meals are widely used as high-protein feed ingredients, reducing reliance on soy-based alternatives and lowering feed costs [61]. In aquaculture-intensive regions such as Southeast Asia, rendered pork and poultry by-products are increasingly used as substitutes for fishmeal, promoting circularity and reducing pressure on wild fish stocks [43,62]. In crop production, blood meal and bone ash serve as nutrient-dense organic fertilizers. Blood meal provides readily available nitrogen for vegetative growth, while bone ash supplies phosphorus and calcium essential for root and fruit development [63]. These materials also enhance soil structure, water retention, and microbial activity, making them valuable in both organic and conventional farming systems.

Despite their potential, valorization efforts face regulatory and technological challenges. For instance, the EU’s risk-based classification system restricts the use of high-risk materials in animal feed following the BSE crisis [30]. Ensuring product safety requires advanced purification technologies such as ultrafiltration for blood proteins capable of removing pathogens and contaminants [64]. Economic feasibility also varies across regions: while small-scale rendering in developing countries often focuses on soaps and lubricant production, biorefineries in the Global North integrate multiple processing streams to produce high-value outputs such as bioplastics and adhesives [51].

Industrial valorization of ABPs reduces landfill volume by up to 90% [38], mitigates environmental impacts, and enhances economic resilience across the meat supply chain. Taken together, these pathways demonstrate a robust model of resource optimization that contributes to food system sustainability, environmental protection, and cross-sector economic growth.

## 4. Consumer Utilization of Animal By-Products Based on Different World Regions

The acceptability of ABPs for human consumption differs widely across global regions shaped by regulatory frameworks, perceived nutritional benefits, economic factors such as per capita income, and deeply rooted cultural and religious beliefs [37,65]. As expressed in the adage “one man’s food is another man’s poison”, by-products considered delicacies in one region may be rejected elsewhere [12]. While some cultures value nose-to-tail consumption, others limit intake to few edible organs, with remaining materials redirected into non-food applications.

During the early 1990s, the value of ABPs declined due to technological advancements that enabled synthetic alternatives for leather, wool, animal fats, and soap [66,67]. However, renewed interest in sustainability has since highlighted the importance of utilizing both edible and inedible by-products to enhance resource efficiency and minimize the environmental impacts associated with expanding meat production [68]. Today, by-products serve as key inputs for agricultural fertilizers and feeds, pharmaceutical products such as gelatin and bioactive peptides, bioenergy production, and functional food development. Increasing utilization efforts focus on converting these materials into traditional foods or nutrient-rich ingredients for human consumption.

Globally, edible offal and blood remain important sources of affordable protein, vitamins, and minerals [38]. However, consumer perception varies greatly between the Global South where direct consumption is widespread and Global North, where processed or indirect forms dominate and human consumption continues to decline [69].

**Figure 3:**
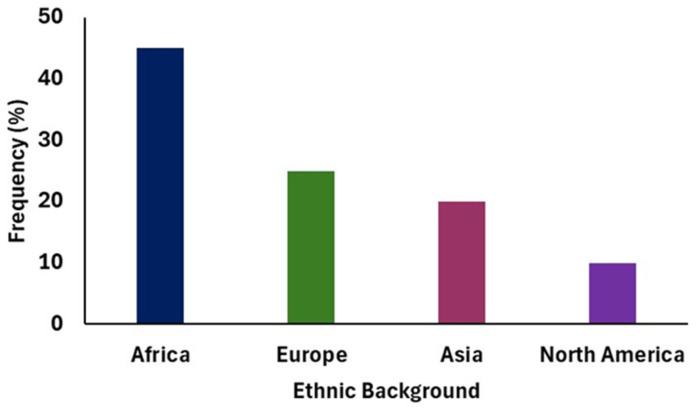
Background distribution of survey respondent.

To better understand these jurisdictional differences, an online survey was conducted across diverse regions, as shown in Figure 3, including Africa (predominantly Nigeria), North America (Canada and USA), Europe (Ireland), and Asia (India, Singapore, Nepal, Korea). The anonymous questionnaire assessed consumption frequency, product preferences, cultural influences, perceived barriers, and motivators alongside demographic variables such as gender, age, ethnicity, and country of residence. The findings provide valuable insights into regional attitudes towards offal consumption and highlight opportunities for promoting sustainable valorization of animal by-products.

### 4.1. Survey Methodology

#### 4.1.1. Study Design

This survey component was designed to complement the literature review by providing primary, up-to-date insights into consumer perceptions across jurisdictions. A cross-sectional, anonymous online survey was conducted to assess consumer perceptions, attitudes, and behaviors regarding the consumption and utilization of slaughterhouse-derived meat by-products (offal). The online format enabled efficient data collection across geographically dispersed populations and facilitated comparison between Global North and Global South regions. Ethical approval for the study was obtained from the University of Alberta Research Ethics Board (protocol code Pro0015033), and the study adhered to established guidelines for survey-based research.

#### 4.1.2. Survey Population and Sampling

A total of 358 respondents participated in the survey. Participants were recruited using convenience sampling through academic networks, social media platforms, and professional forums. The sample included individuals from Africa (predominantly Nigeria), North America (Canada and the United States), Europe (Ireland), and Asia (India, Singapore, Nepal, and Korea). Eligibility criteria required participants to be 18 years or older and to have prior experience or familiarity with meat consumption.

#### 4.1.3. Survey Instrument and Structure

The questionnaire was structured into five sections: (i) demographic characteristics (age, gender, ethnicity, and country of residence), (ii) consumption patterns (frequency of meat and offal consumption and preferred species), (iii) culinary practices (preparation methods and consumption formats), (iv) perceptions and barriers (sensory attributes, safety concerns, and cultural acceptance), and (v) behavioral drivers and attitudes (willingness to consume offal, sustainability awareness, and climate change perceptions). The survey included closed-ended and multiple-response questions. Perception-based responses were measured using a 5-point Likert scale (1 = strongly disagree to 5 = strongly agree), while categorical variables were used to assess consumption patterns and preferences. The questionnaire is provided as a Supplementary Materials.

#### 4.1.4. Data Collection

Data were collected electronically using an online survey platform over a defined study period. Participation was voluntary, and informed consent was obtained from all respondents prior to survey completion. No personally identifiable information was collected, ensuring anonymity and confidentiality.

#### 4.1.5. Data Processing and Analysis

Data were exported to Microsoft Excel for cleaning and coding and subsequently analyzed using SAS software (SAS 9.4 (Institute Inc., Cary, NC, USA)). Descriptive statistics, including frequencies and percentages, were calculated for all categorical variables. Multi-response questions were coded as binary variables (1 = selected; 0 = not selected). Where appropriate, responses were interpreted in the context of regional differences to support jurisdictional comparisons.

### 4.2. Meat By-Product Consumption in the Global South

In the Global South, including much of Africa, South America, and developing regions of Asia, ABPs play an essential role in food security. ABPs offer nutrient-dense, affordable protein sources in areas where access to premium cuts is limited. Consumption is driven by culinary traditions, minimal waste cultural philosophies, and economic necessity. The survey results corroborated this trend: 89.9% of respondents (*n* = 358) reported consuming offal, with especially high acceptance among First Nations/Indigenous groups (100%), and African participants (93.38%), compared to lower acceptance in parts of Europe (34.78%).

Similar patterns have been reported in independent studies. In Spain, consumer openness towards offal varies by attitudinal segments, with factors such as health-conscious skepticism and reluctance influencing purchase intent [70]. In Italy, food neophobia and disgust sensitivity reduce willingness to purchase beef offal [71]. Conversely, increased familiarity and information can enhance acceptance of novel co-product ingredients, as noted among Irish consumers [72]. The following subsections provide regional details.

#### 4.2.1. Africa

In Sub-Saharan Africa, edible offal forms constitute a sizable portion of total animal protein intake. Offal such as liver, kidney, tripe, lungs, tongue, and feet are widely incorporated into stews, soups, grilled dishes, and ceremonial meals. Local delicacies, such as *ponmo* (Nigeria), *wele* (Ghana), and *kanda* (Cameroon), are prepared from processed hides and are consumed regularly [73]. In South Africa, growing meat demand has increased the consumption of tripe and intestines in informal markets, with an estimated per capita intake of 13 kg of offal [74].

**Figure 4:**
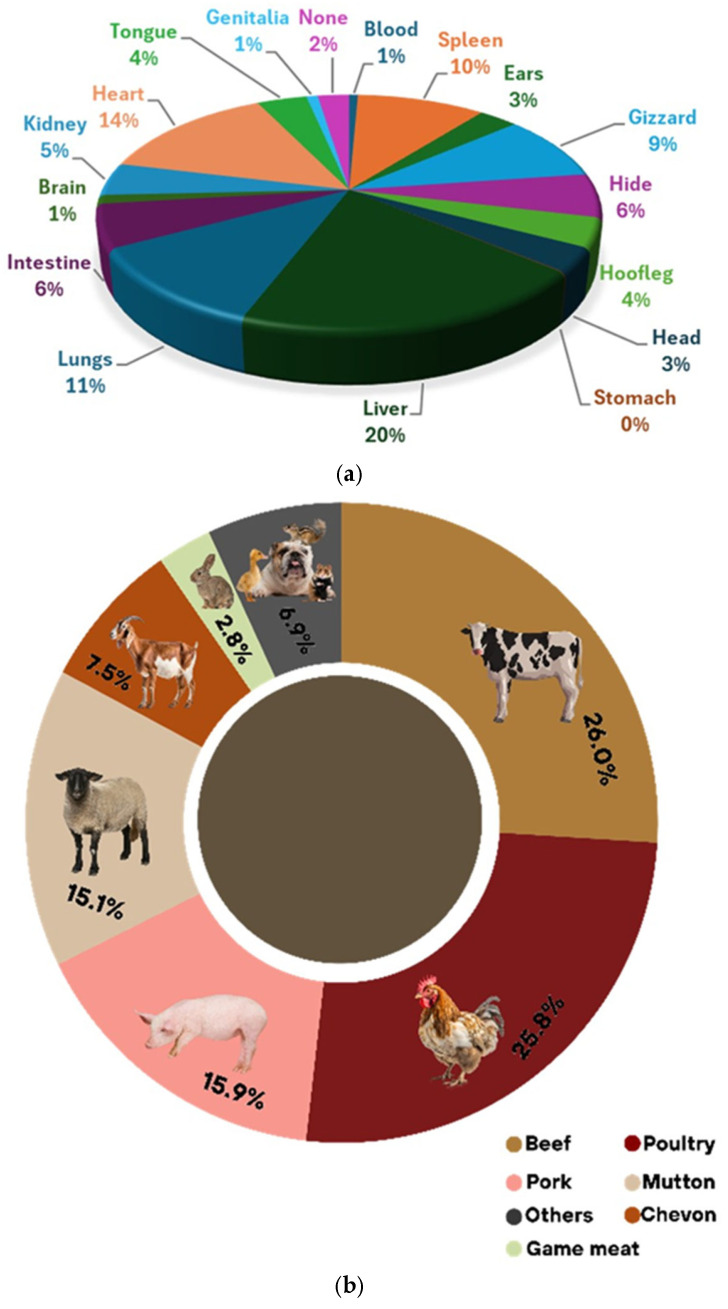
(**a**) Type of offal consumed by survey respondents. (**b**) Type of meat consumed by respondents.

Survey responses (Figure 4a,b) mirrored regional patterns and type of meat consumed: the most consumed cuts were liver (19.7%), heart (14.0%), and lungs (11.3%), and gizzard (9.1%), reflecting strong cultural integration. However, safety concerns persist due to informal slaughter practices and potential environmental contamination. Urbanization has also led to rising demand for processed imports, though cultural festivals continue to reinforce traditional consumption [75]. Examples of traditional dishes prepared from ABPs across regions are summarized in Table 3. To reflect the global diversity in the utilization of meat by-products, examples of traditional dishes across multiple regions are presented in Table 3.

#### 4.2.2. South America

In South America, traditional dishes such as *mocotó* (cow’s foot stew) in Brazil and mondongo (tripe soup) in Argentina and Uruguay demonstrate long-standing cultural acceptance [76]. Livestock abundance and affordability make by-products an important protein source for low-income populations [77,78]. Brazil’s Ministry of Agriculture reports that approximately 15% of beef yield (including offal and secondary cuts) is directed towards domestic offal markets, while high-value cuts are prioritized for export [79]. Survey data showed that consumers commonly preferred offal in soups/sauces (35.6%) and roasted/grilled preparations (33.7%), indicating that texture- and flavor-modifying cooking methods enhance acceptability. Argentina’s asado tradition incorporates sweetbreads and blood sausages, reflecting strong culinary integration despite modern dietary shifts [80]. However, increasing urbanization and waste management issues underscore the need for improved valorization infrastructure [81].

#### 4.2.3. Developing Region of Asia

Across South and Southeast Asia, offal remains a dietary staple rooted in cultural, economic, and religious practices. In India, consumption of ABPs varies significantly across communities due to regional customs and religious restrictions. Organ meats such as liver, kidneys, heart, and brain are key ingredients used in dishes such as *Kaleji Masala, Gurda Kaleji*, and *Maghaz Masala* [82]. Tripe, stomach, and intestines are prepared in dishes like Paya Curry and *Katakat* [83,84], while feet (trotters) and tongue are incorporated into slow-cooked stews [85].

Offal consumption across Asia is not solely cultural but also economically motivated, providing affordable protein for low-income households [86]. Dishes such as Pakistan’s katakat (mixed organ stir-fry) and the Philippines’ dinuguan (pork blood stew) reflect diverse culinary integration [75]. Indonesia alone produced more than 1.2 million tons of offal in 2021, with over 130 traditional dishes reported across 23 provinces [87]. In Vietnam and Thailand, intestines and other offal cuts feature in dishes such as pho and sai ua sausages, with per capita by-product consumption estimated at 5–10 kg [75,88]. Recent innovations include the incorporation of nano-calcium derived from eggshells to enhance nutritional and physical properties of offal-based sausages [89,90].

Religious dietary laws also play a significant role in shaping the consumption and utilization of animal by-products across different regions. In Hindu communities, the consumption of beef and, in some cases, blood-derived products is restricted. Islamic dietary laws prohibit the consumption of non-halal organs and blood products. Similarly, in Judaism, kosher dietary laws strictly forbid the consumption of blood and require specific slaughter and processing practices [87]. In Christianity, although dietary restrictions are generally less prescriptive, certain denominations discourage or prohibit the consumption of blood based on biblical interpretations (Acts 15:20). These religious frameworks collectively influence regional preferences and determine which by-products are accepted within the food chain.

### 4.3. ABP Consumption in the Global North

Industrialized nations exhibit lower direct consumption of by-products compared to the Global South. Cultural preferences, stringent food safety regulations, and market dynamics have shifted utilization toward rendering, pet food manufacturing, and industrial applications such as biofuels and fertilizers [38]. Human consumption persists in traditional dishes but remains limited overall.

#### 4.3.1. Europe

In Europe, many countries retain traditional uses of liver, tongue, kidney, and tripe in regional cuisines, particularly in Romania, Turkey, Spain, Bulgaria, and the United Kingdom [37]. Blood is used in products such as blood sausage, pudding, and bread [91], supported by a commercial network for food-grade blood proteins [16]. Blood-enriched products have also been used to address iron deficiency anemia among school-aged children [92]. Additionally, meat products fortified with bovine blood have been employed to treat iron deficiency in school children with *ferropenic* anemia across Europe, displaying their potential to address nutritional deficiencies, while intestines are valued for their texture.

**Figure 5:**
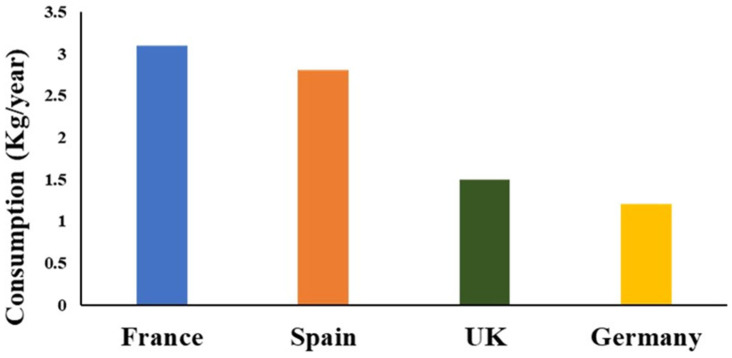
Estimated per capita consumption of edible offal in selected European countries (2022). Source: [2].

**Figure 6:**
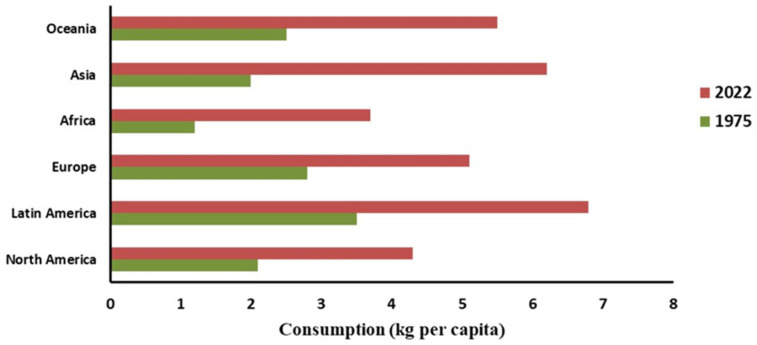
Regional per capita consumption of meat by-products (1975 vs. 2022). Source: [2,3,68,94].

Despite cultural heritage, overall consumption has declined, with 51% of Europeans reporting reduced intake [88]. At the same time, industry-driven valorization is increasing, particularly through the development of functional foods, nutraceuticals, protein hydrolysates, and biodegradable materials [93]. Adoption of enzymatic hydrolysis, nanotechnology, automation, and blockchain-based traceability further supports EU sustainability goals. Per capita consumption in selected European countries is shown in Figure 5, while Figure 6 illustrates the change in trend for global regional per capita consumption of meat by-products between 1975 and 2022.

#### 4.3.2. Developed Asia

Asia represents a major market for imported offal from Europe, driven by strong culinary traditions and high demand for specialty cuts [44]. In Japan, rigorous food safety regulations support the safe consumption of liver, heart, and other organs, especially in dishes such as yakiniku. Japan’s spongiform encephalopathy (BSE) mitigation protocols have significantly reduced safety concerns related to offal imports.

In China, offal such as tripe, intestines, and kidneys are staples in hot pot, braises and stir-fries, reflecting a cultural emphasis on minimizing waste and using all parts of the animal [95]. Large population size further drives demand for value-added by-product-derived products [96]. Similarly, in Korea, organ meats are an essential component of traditional cuisine, appearing in dishes such as barbecue, soups, and sausages including processed innovations using plant binders to improve texture and flavor [97]. While urban consumption is declining, traditional celebrations sustain demand [98].

#### 4.3.3. North America

In the United States and Canada, retail offal consumption is limited, with most by-products directed into pet food or exported to markets in Asia, Latin America, and Africa [5,99]. However, immigrant communities sustain niche markets for beef tongue, pork liver, tripe, and chicken feet [67]. The USDA ERS [68] reports that over 50% of edible beef offal is exported, particularly to Mexico, Egypt, and the Philippines. Although direct consumption remains low, interest in nutrient-dense foods is gradually increasing. The North American edible offal market is projected to grow at 4.0% CAGR between 2023 and 2030, driven by rising pet food demand and incorporation of offal into functional and processed foods [100,101]. In Canada, specific communities such as French Canadians and Indigenous groups preserve cultural practices involving organ meats, including liver plate and consumption of moose or caribou organs [102]. Canadian researchers are also exploring new bioprocessing applications such as bio-based flocculants and phosphate alternatives for meat enhancement [103,104]. For small- and medium-sized meat processors, composting offers a sustainable disposal method for bones, trimmings, and other co-products. Composting technologies including windrow, aerated static pile, in-vessel, and vermicomposting can reduce waste volumes by up to 30% while generating nutrient-rich soil amendments [105]. Challenges such as odor, pathogen control, and regulatory compliance remain but can be mitigated through proper management [106].

Across the Global North, lower direct consumption of ABPs contrasts sharply with high cultural integration seen in many regions of the Global South. Instead, northern systems rely heavily on export markets and non-food valorization pathways. Table 4 provides a comparative overview of consumption patterns across world regions, while subsequent sections explore additional opportunities for advancing sustainable utilization.

## 5. Potential Future Utilization or Valorization Opportunities

The growing emphasis on sustainability, waste reduction, and circular bioeconomy models is reshaping how meat by-products are perceived and utilized globally. While conventional applications such as rendering, feed manufacture, and fertilizer production continue to dominate, emerging technologies are creating opportunities for high-value utilization across food, health, materials science, and energy sectors. As global meat production continues to rise, the demand for innovative valorization approaches will intensify. However, the scope and feasibility of these opportunities differ markedly between the Global South and Global North due to variations in technological capacity, regulatory frameworks, consumer acceptance, and market infrastructure. The following sections present a comparative review of potential valorization pathways in both regions.

### 5.1. Valorization Potentials in Global South

#### 5.1.1. Nutraceuticals and Functional Ingredients

In the Global South, particularly in Africa, nutraceutical development from meat by-products is gaining attention as a strategy to address chronic malnutrition and micronutrient deficiencies. Fermentation-based hydrolysis using proteolytic strains such as Lactobacillus rhamnosus has shown strong potential for converting nutrient-rich offal into bioactive peptides [107]. These peptides, including antioxidant compounds derived from lung elastin and antihypertensive fractions from liver hydrolysates, have demonstrated functional benefits suitable for fortified food applications [108]. Importantly, local peptide production could reduce reliance on imported supplements while stimulating rural value chains and unlocking new livelihood opportunities.

#### 5.1.2. Small-Scale Collagen and Gelatin Production

Collagen and gelatin extraction represent another promising avenue for high-value utilization in South and Southeast Asia as well as Africa. In regions such as India, Indonesia, and Vietnam, the adoption of enzymatic extraction and freeze-drying technologies has enabled the production of food-grade and cosmetic-grade collagen with enhanced amino acid profiles [109]. In Africa, pilot-scale projects in Nigeria and Ethiopia have shown that goat and cattle hides can be efficiently converted into gelatin suitable for confectionery and pharmaceutical capsule production [10,110]. The use of solar-assisted drying offers additional benefits by lowering energy costs, making collagen processing economically viable for small and medium enterprises. This pathway adds value to materials traditionally regarded as low-worth, supporting local cosmetic and wellness industries.

#### 5.1.3. Blood Protein Fortification

Blood remains an underutilized yet high-potential resource in the Global South. Slaughterhouse blood rich in heme iron and essential amino acids can be transformed into functional ingredients through advanced fractionation and enzymatic hydrolysis [107]. Resulting products such as hame iron-fortified hydrolysates exhibit high antioxidant activity and improved bioavailability, making them ideal for addressing widespread iron-deficiency anemia, affecting up to 47% of African children and 52% of children under five [111]. Applications include fortified flour, infant weaning formulations, and nutrition products for pregnant and elderly populations. Evidence from Le Thi Hong [112] demonstrates significant improvements in hemoglobin levels following consumption of blood-protein fortified foods. While cultural acceptance remains a barrier, culturally sensitive product development and clear labeling strategies are essential for mainstream adoption. As processing technologies advance, blood-derived ingredients could offer scalable and sustainable solutions to micronutrient deficiencies.

#### 5.1.4. Bioenergy from Slaughterhouse Waste

Bioenergy recovery from slaughterhouse waste is emerging as a major sustainability opportunity in the Global South. Anaerobic digestion of gut contents, dissolved air flotation sludge, and blood have shown significant potential for biogas production. South American and African pilot projects demonstrate that co-digestion with municipal solid waste enhances methane yields, reduces landfill dependence, and mitigates environmental pollution [109,113]. The FAO [79] reports that a 500 m^3^ digester in Kenya can offset up to 60% of the electricity needs of a medium-sized abattoir, illustrating the strong alignment of biogas systems with circular economy principles and SDGs. Table 5 provides an overview of priority valorization opportunities identified for the Global South region.

### 5.2. Valorization Potentials in Global North

In contrast to the Global South region, where valorization efforts often focus on nutrition security and small-scale processing, the Global North is advancing high-technology, capital-intensive, and sustainability-driven valorization pathways. These emerging sectors span valorization potentials in the Global North across biorefining, precision fermentation, biomedical engineering, and green chemistry.

#### 5.2.1. Biorefinery Integration

Biorefineries are becoming central to the modernization of ABP utilization in the Global North. Through advanced rendering and fractionation technologies, ABPs are converted into sterile protein meals, high purity fats, bioactive peptides, and mineral concentrates that serve as feedstock for biodiesel, oleochemicals, fertilizers, and functional foods [56]. EU-funded platforms such as PROLIFIC and ELLIPSE demonstrate near-zero waste refining of offal streams, including the co-processing of slaughterhouse waste with paper sludge to produce biodegradable plastics such as polyhydroxyalkanoates (PHAs), for used in packaging and agriculture [3,114].

#### 5.2.2. Precision Fermentation and Cultured Ingredients

Precision fermentation is redefining alternative protein production by leveraging AI-driven microbial platforms to convert waste-derived amino acids into high-value compounds such as flavoring agent, enzymes, and microbial proteins. This process can achieve up to 90% lower GHGEs than conventional livestock production [115]. Offal hydrolysates provide a cost-effective nitrogen source for fermentation systems producing ingredients used across food and biotech industries [116].

#### 5.2.3. Microbial Protein Production

Microbial protein derived from slaughterhouse co-streams offers a sustainable alternative to traditional protein sources. Fermenting agricultural residues and meat industry wastes yields mycoproteins and bacterial biomass with high nutritional density and significantly reduced environmental impacts [117]. These microbial proteins are increasingly used in cultivated meat media and hybrid protein products, supporting broader food system resilience [115].

#### 5.2.4. Biomedical Scaffold Production

Biomedical and cultured meat sectors are increasingly adopting animal-derived scaffolds to mimic the extracellular matrix. Tissues such as bovine pericardium and porcine dermis are processed into medical-grade collagen used in tissue engineering, wound dressings and regenerative medicine [33]. This sector is expanding rapidly, with collagen-based biomaterials projected to reach USD 5.6 billion by 2030 [52]. These scaffolds also contribute to improving structural integrity and nutritional properties in cultured meat production.

#### 5.2.5. Green Chemistry and Bioplastics

Meat by-products are gaining traction as feedstocks in green chemistry, particularly in the production of biodegradable plastics and bio-based polymers. Rumen content, belly grass, hair keratin, and hide-derived collagen can be converted into PHAs, hydrogels, films, and other sustainable materials [57]. These innovations align strongly with EU circular bioeconomy goals and North American sustainability targets [50,61,114], while also reducing landfill burden and improving water and soil quality.

Overall, these valorization pathways indicate a strategic shift in the Global North toward high-tech, low-waste, and environmentally conscious systems capable of creating new economic value across food, energy, and biomedical sectors.

### 5.3. Consumer Perception on Alternative Uses and the Valorization of ABPs

Survey findings presented in Section 4 provide the methodological basis for interpreting global consumer attitudes toward the utilization and valorization of animal by-products. Building on these results, respondents from diverse regions, including Africa, North America (Canada and the United States), Europe (Ireland), and Asia (India, Singapore, Nepal, and the Republic of Korea), among others, demonstrated varied perspectives on alternative utilization pathways for offal.

Respondents showed clear support for non-food valorization pathways, including pharmaceutical and cosmetic extraction (12.1%), animal feed production (20.0%), fertilizer use (20.0%), and energy generation (15.3%). These preferences align with established global trends documented in the literature, where collagen and gelatin extraction, bioactive peptide production, rendering, pet food manufacture, and bioenergy pathways are dominant [12,16,23,38].

**Figure 7:**
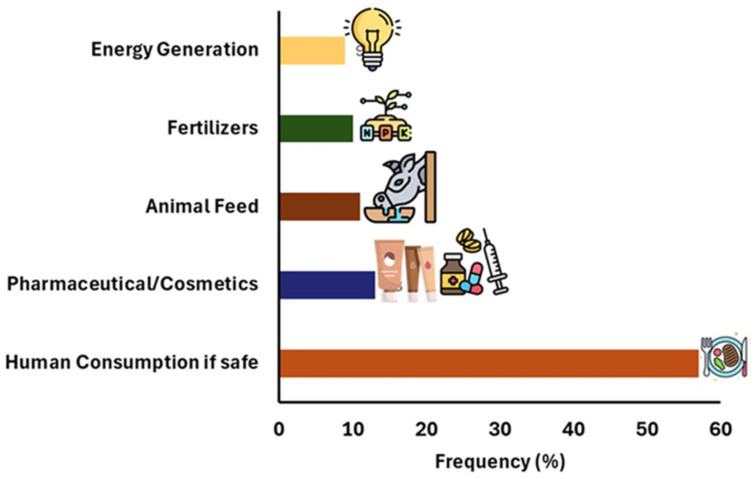
Consumer perception on alternative use of meat by-products.

Economic analyses further demonstrate that edible by-products provide the highest unit value, followed by pet food ingredients, while rendering remains as a lower-value outlet constrained by hygiene and processing costs [41]. Recent reviews highlight that expanding the human-food pathway is strategically beneficial where cultural acceptance and safety standards can be secured [14,72]. These insights are consistent with consumer responses observed in the present survey has shown in Figure 7.

#### Jurisdictional Opportunity Space

Synthesizing survey findings with the existing literature reveals three immediate opportunity areas for enhancing jurisdictional utilization of ABPs: (i) scaling edible valorization by targeting high-acceptance cuts such as liver and developing hybrid foods (e.g., micronutrient-fortified burgers), supported by compositional evidence and clear safety–nutrition claims [16,70]; (ii) adopting stepwise utilization hierarchies, particularly where edible channels are limited, using an approach that prioritizes intermediate streams such as pet food production, collagen extraction, and enzyme recovery before diverting residuals to bioenergy to maximize value capture [12,95]; and (iii) implementing behavioral and policy interventions, including eco-labeling, procurement incentives, regulatory clarity on labeling and trade, and public awareness programs to normalize edible by-product consumption [3,90,92]. Collectively, these strategies support economic viability and cultural acceptance while aligning with broader circular bioeconomy and sustainability goals across jurisdictions.

## 6. Conclusions

Animal by-products constitute a substantial proportion of live animal weight of up to 40–60% and represent significant nutritional, economic, and functional value across global food systems. Yet their utilization and valorization remain uneven across jurisdictions. In the Global South, edible offal and other co-products are deeply embedded in traditional diets, serving as affordable sources of protein and micronutrients. Conversely, in the Global North, direct consumption is comparatively limited, and by-products are redirected to rendering, pet food production, and industrial applications, shaped by stringent safety regulations, consumer perceptions, and market structures. Across all regions, consumer acceptance emerges as a crucial factor influencing edible use: cultural familiarity, culinary skill, sensory expectations, and confidence in safety heavily shaped willingness to consume these products.

Findings from the present survey reinforce this pattern. While disgust was not the primary deterrent among non-consumers, unfamiliarity (20.3%), lack of culinary skills (15.3%), and texture or taste concerns (13.6%) were key barrier patterns that mirror findings from Europe, where concerns about “undesirable compounds,” neophobia, and sensory attributes frequently limit acceptance [70,71]. Importantly, environmental values also influence consumer choices. Survey data indicated that 96.6% of respondents believed human activities drive climate change, and 45.16% expressed increased willingness to consume edible co-products once informed that doing so reduces food waste and supports climate mitigation, aligning with research showing that eco-labeling and behavioral nudges can steer consumers toward environmentally beneficial foods without challenging identity-related habits [16,90].

These findings underscore that optimizing the utilization of animal by-products requires a multi-level strategy that integrates technological innovation, regulatory clarity, and targeted consumer education. In the Global South, development of small- to medium-scale processing infrastructure including facilities for gelatin extraction, blood plasma recovery, collagen production, and biogas generation will be essential for strengthening local value chains. In contrast, the Global North is well positioned to scale biorefinery-based systems capable of fully integrating protein, lipid, and mineral streams into high-value applications. Strengthening supply-chain linkages that connect small and medium enterprises to these advanced valorization outlets would further improve resource efficiency across jurisdictions.

Consumer-focused interventions remain equally important. Product reformulation that incorporates offal into familiar formats such as sausages, pâtés, fortified flours, hybrid burgers, and protein powders can mitigate sensory and familiarity barriers, as supported by studies highlighting the positive impact of information provision, nutritional framing, and familiarity effects on consumer acceptance of co-products [16,70,72]. Clear communication about nutritional and safety benefits, combined with culinary education and recipe development, is likely to increase acceptance in populations where direct consumption is low. Moreover, harmonized standards for edible by-product processing, improved traceability, and expanded hygienic training programs for abattoir and processing workers will enhance both domestic and international confidence in by-product safety.

Finally, research, innovation, and cross-continental collaboration will be critical for advancing sustainable valorization. Open access knowledge platforms, partnerships for technology transfer, and support for pilot-scale demonstration projects can help adapt advanced processes such as enzymatic hydrolysis, precision fermentation, collagen extraction, and biogas recovery to diverse resource settings. These approaches collectively reinforce the role of animal by-products as valuable components of a circular bioeconomy and sustainable global food system.

In summary, the strategic valorization of ABPs offers substantial opportunities to reduce waste, improve environmental performance, strengthen food and nutrition security, and create new economic pathways across regions. Achieving these outcomes will require coordinated action across policy, industry, and consumer domains, integrating technological capacity, regulatory alignment, and behavioral insights to ensure that animal by-products contribute optimally to sustainable meat production and the global bioeconomy.

## Figures and Tables

**Table 1:** Classification of slaughterhouse-derived meat by-products.

Category	Sub-Category	Examples	Primary Applications
Edible by-products (Offal)	Organ meats	Liver, kidney, heart, brain	Direct human consumption, traditional dishes
	Gastrointestinal components	Tripe, intestines	Food products, natural casings
	Blood and derivatives	Blood, plasma	Food ingredients, binders, nutritional products
	External parts	Skin (processed), tongue	Culinary uses, specialty dishes
Inedible by-products	Structural materials	Bones, horns, hooves	Gelatin, fertilizers, animal feed
	Hides and skins	Raw hides	Leather, collagen extraction
	Rendering materials	Fat trimmings, meat residues	Tallow, biodiesel, pet food
	Waste streams	Specified risk materials	Energy (biogas), composting

Note: Edible by-products are classified as meat under EU Regulation 853/2004, while inedible by-products are regulated under EC Regulation 1069/2009.

**Table 3:** Examples of traditional dishes incorporating meat by-products across regions.

Region	Country/Area	Dish Name	By-Product Used	Description
**Africa**	Nigeria	Nkwobi	Cow foot (skin)	Spicy delicacy made from cow skin
	South Africa	Mala Mogodu	Tripe	Tripe stew commonly consumed
**Europe**	Scotland	Haggis	Liver, heart, lungs	Traditional dish mixed with oats and spices
	France	Foie gras	Liver	Processed duck/goose liver delicacy
**Asia**	Philippines	Dinuguan	Blood	Savory stew made with pork blood
	China	Braised Intestines	Intestines	Popular street and home dish
**Latin America**	Mexico	Menudo	Tripe	Soup traditionally consumed during celebrations
	Peru	Anticuchos	Heart	Grilled skewered beef heart
**Middle East**	Turkey	Kokoreç	Intestines	Seasoned grilled intestines
**Global/Industrial**	Multiple	Sausages	Intestines (casings)	Widely used in processed meat products

**Table 4:** Comparative overview of consumer utilization patterns by region.

Region	Offal Acceptance	Main Products Consumed	Key Market Drivers	Constraints
Africa	High	Tripe, liver, hides, trotters	Cultural heritage, affordability	Food safety, informal sector practices
Europe	Moderate–High	Blood sausages, tripe, liver	Culinary tradition, artisanal gastronomy	Regulatory cost, generational decline
Asia (Developing)	High	Liver, kidney, blood dishes	Tradition, minimal waste ethos	Religious dietary restrictions
Asia (Developed)	High	Grilled offal, intestines	Culinary culture, premium restaurant sector	Stringent safety protocols
North America	Low	Tongue, chicken feet (ethnic)	Immigrant markets, exports	Consumer aversion, regulation
South America	Moderate–High	Tripe soups, feet, blood dishes	Tradition, low-cost protein	Urban dietary shifts

**Table 5:** Priority valorization pathways for the Global South.

Pathway	Example By-Products	Potential Products	Key Benefits
Nutraceutical peptides	Liver, lungs, heart	Functional food powders	Added value, health impact
Community gelatin plants	Hides, skins, bones	Gelatin, collagen supplements	Job creation, import substitution
Blood fortification	Blood plasma	High-iron flour, biscuits	Nutritional security
Biogas production	Paunch content, blood	Electricity, heat	Reduced energy cost, lower emissions

## Data Availability

The datasets presented in this article are not readily available due to privacy restriction from ethical approval.

## Supplementary Materials

The following supporting information can be downloaded at: https://www.mdpi.com/article/10.3390/foods15081324/s1, The survey questionnaire used for data collection for this study is available as supplementary material attached to this manuscript.
