# Supplementary material for: Jurisdictional Comparison in the Utilization and Valorization of Animal By-Products of Slaughterhouse-Origin: A Global Review
Source: Foods. 2026 Apr 10;15(8):1324. doi: 10.3390/foods15081324 (PMC13114427; doi:10.3390/foods15081324)
Supplement: Supplementary file 1 [file foods-15-01324-s001.zip › foods-4222392-supplementary.pdf]

UNIVERSITY OF ALBERTA

CONSUMER PERCEPTION OF CONSUMING MEAT CO-PRODUCTS/OFFAL

Dear Respondent,

This survey is a component of my research study at the University of Alberta. I will appreciate your candid opinion and participation in this survey. The survey takes **approximately 7 mins** to complete.

Introduction: Meat co-products refer to the parts of an animal body or products derived from the entire body of an animal, other than the carcass. The meat industry generates many of these co-products during the harvesting process. These may include "variety meats" (intestine, liver, tongue, lungs, heart, kidney), heads, bones, carcasses, blood, skin, viscera, hooves, and feathers, among others.

Purpose: This survey is part of a research project focusing on the valorization of meat co-products. It aims to assess consumer perception of edible meat co-product consumption either in their original state or conversion to other products. Your voluntary participation is of utmost importance in helping generate feedback and input, to develop the appropriate model(s) for the human utilization of meat co-products based on the data to be generated from this survey, thereby reducing their environmental and economic effect.

Data Protection: All data collected from this survey are subject to your voluntary participation, and responses are collected anonymously, securely stored, and protected. Data collected are only for research purposes. Results from this study may be published in a report but no identifiable data collected from this survey will be included.

Confidentiality and Anonymity: The information that you will share will remain strictly confidential. Anonymity is guaranteed. However, while email address will be collected if you want to participate in the survey raffle draw, it will be collected separately and will not be linked to your survey response it will be anonymous. If at any point in the research you do not wish to continue or want your data to be deleted, please contact ifedayo@ualberta.ca.

Risk: There's no known risk associated with this survey

Voluntary Participation: You are under no obligation to participate and if you choose to participate, you may refuse to answer questions that you do not want to answer.

Participation Withdrawal: Should you choose to withdraw midway through the electronic survey, simply close the link and no responses will be included. However, given the anonymous nature of the survey, once you have submitted your responses it will no longer be possible to withdraw your data from the study.
Contact Information: If you have any questions about the research now or later, please contact the study principal investigator **Ifedayo** via **ifedayo@ualberta.ca.** If you have any questions regarding your rights as a research participant, you may contact the University of Alberta Research Ethics Office at ethics@ualberta.ca or +1(780)492-2615 and quote Ethics ID Pro00150335. This office is independent of the researchers.

Participation Incentive: To appreciate participants for completing this survey, respondents have the option to **voluntarily participate in a raffle draw and win one of six $25 or equivalent prize with a winning odd of 6 out of 500** the survey. Winners will be contacted via the email provided in the raffle draw section of this survey.

NB: The email address is only for raffle draw purposes and participation is optional. This email address will not be linked to your survey responses.

[illegible]

Participant Consent

Proceeding to the next section of this survey affirms that you have read the information provided above and consent to your voluntary participation in this survey. You understand that this survey's data are for research purposes only and are protected and your consent is provided by agreeing here and submission of the survey.

Have you read the survey information provided above and consent to your voluntary participation in this survey? *

- ◯ Yes, I have read and understand that my consent is provided by agreeing here and submission of the survey.
- ◯ No, I do not consent to participate

RAFFLE DRAW

To appreciate participants for completing this survey, respondents have the option to ***voluntarily participate in a raffle draw to win one of six $25 or equivalent prize with a winning odd of 6 out of 500*** *in the survey. You will be directed to a separate web page where you can input your contact information to enter into the draw and your contact information will not be associated with your survey responses. Winners will be contacted via the email provided in the raffle draw section of this survey.* ***Click on this link https://forms.office.com/r/7gseXMjkz5 to participate in the raffle drawNB: This email is collected for the purpose of raffle draw only, if you don't wish to participate in the raffle draw, kindly skip this section***

Email Address

Survey Questions

Do you normally consume meat and/or meat products *

- ◯ Yes
- ◯ No

How often do you consume meat (Please select one)? *

- ◯ More than once a day
- ◯ Once a day
- ◯ 2-3 times a week
- ◯ Once a week
- ◯ Rarely
- ◯ Never

Which of these meats do you eat (Please select all that applies)? *

- [ ] Beef
- [ ] Poultry (chicken/turkey/duck)
- [ ] Pork
- [ ] Mutton (adult sheep/ram)
- [ ] Lamb (young sheep)
- [ ] Chevon (goat meat)
- [ ] Veal (meat of calves)
- [ ] Game meat (deer, elk, moose, rabbit, etc)
- [ ] Carabeef (buffalo meat)
- [ ] Prefer not to say
- [ ] Other

Do you eat meat co-products/offal from meat animals? *

- ○ Yes
- ○ No
- ○ Prefer not to say

If you eat meat co-products/offal, which animals do you normally get them from (Please select all that apply)? *

- ☐ Cattle
- ☐ Pig
- ☐ Poultry
- ☐ Sheep/Ram
- ☐ Goat
- ☐ Game meat (deer, elk, moose, rabbit etc.)
- ☐ Prefer not to say
- ☐ Other

From these animals, which meat co-products/offal do you eat (Please select all that apply)? *

- [ ] Liver
- [ ] Heart
- [ ] Spleen
- [ ] Lungs
- [ ] Kidney
- [ ] Intestines
- [ ] Ears
- [ ] Genitals
- [ ] Blood
- [ ] Hide/Skin
- [ ] Hoof/Legs/feet
- [ ] Brain
- [ ] Tongue
- [ ] Gizzard
- [ ] Head
- [ ] Prefer not to say
- [ ] Other

In what form do you consume these meat co-products/offal? (Type your answer below, e.g. in soups, rice, sauces etc.) *

If you don't eat meat co-products/offal from meat animals, please can you specify why you don't (select all that applies)? *

- [ ] I eat them always in different cuisines
- [ ] I don't mind eating them, I just don't know where to buy them
- [ ] I don't know how to cook and make them delicious
- [ ] I am afraid, they can make me sick
- [ ] I am not familiar with these types of meat
- [ ] I just hate the taste/flavour
- [ ] They are a waste products, they should not be eaten
- [ ] They are just awful
- [ ] They contain some unhealthy components (like heavy metals), it's dangerous to eat them
- [ ] Why eat them when I can eat real muscle meat?
- [ ] Prefer not to say
- [ ] Other

If you don't eat these meat co-products/offal, what do you think they should be used for (Please select all that applies)? *

- [ ] I normally eat them
- [ ] We should all eat them provided they are safe
- [ ] Feed them to animal (if and when possible)
- [ ] Send them to countries where they eat them
- [ ] Use them as organic manure/fertilizer
- [ ] Use them for energy generation (e.g. biodiesel, biogas, etc.)
- [ ] Extract important components from them for pharmaceutical, cosmetic or functional food applications, if possible
- [ ] Throw them away, they belong in the landfill
- [ ] I don't care
- [ ] Prefer not to say
- [ ] Other

Do you believe in the impact of human activities on climate change? *

- ○ Yes
- ○ No
- ○ Maybe

What could make you consider including these meat co-products/offal in your diet *

- [ ] I already include them in my diet and have no reservation eating them
- [ ] I can eat them if I know they are safe
- [ ] I can eat them if I have the culinary skills to make them delicious
- [ ] If protein powder can be derived from them, I can try them as protein source in my cooking/baking
- [ ] I can try to eat if they are made into burger or sausages
- [ ] If they are added in small proportion in meat or food formulations
- [ ] I will never eat them in any form
- [ ] Prefer not to say
- [ ] Other

Including these co-products/offal in your diet can reduce food waste and have a positive impacts on climate change, will this understanding influence your decision to consume meat co-products/offal? *

- ○ Yes
- ○ No
- ○ Maybe

Please select what applies to you *

- ◯ Male
- ◯ Female
- ◯ Prefer not to say
- ◯ Other

Please select your age group *

- ◯ Below 18
- ◯ Between 18 and 24
- ◯ Between 25 and 34
- ◯ Between 35 and 44
- ◯ Between 45 and 54
- ◯ Between 55 and 64
- ◯ 65 and above
- ◯ Prefer not to say

Which of the following best describes your ethnic background? *

- ◯ African
- ◯ European
- ◯ East Asian
- ◯ South Asian
- ◯ South East Asian
- ◯ First Nations / Indigenous
- ◯ Hispanic or Latin
- ◯ Middle Eastern
- ◯ Prefer not to say
- ◯ Other

What is your country of residence? *

Do you wish to submit your response or withdraw from the survey?
NB: This is the last point you can withdraw from this survey.
Please note that submitting or withdrawing your data will not affect your raffle draw entry *

- ◯ **Yes, I want to submit**
- ◯ **No, I want to withdraw**

This content is neither created nor endorsed by Microsoft. The data you submit will be sent to the form owner.

Microsoft Forms
